# Cities as Evolutionary Islands: Colonization of Isolated Urban Habitats Promotes Immunogenetic Differentiation in a Common Waterbird

**DOI:** 10.1111/eva.70363

**Published:** 2026-11-11

**Authors:** Piotr Minias, Joanna Drzewińska‐Chańko, Jan Jedlikowski, Amelia Chyb, Radosław Włodarczyk

**Affiliations:** ^1^ Department of Biodiversity Studies and Bioeducation, Faculty of Biology and Environmental Protection University of Lodz Lodz Poland; ^2^ Faculty of Biology, Biological and Chemical Research Centre University of Warsaw Warsaw Poland

**Keywords:** birds, immunogenetics, MHC, population differentiation, toll‐like receptors, urbanization

## Abstract

Animals that colonize urban landscapes encounter novel selective pressures, including exposure to altered pathogen communities. While these novel selection regimes may promote adaptation, genetic differentiation between urban and non‐urban populations may also be driven by demographic history and reflect neutral processes, such as drift. Here, we investigated patterns of immunogenetic differentiation among four pairs of urban and non‐urban populations of a common waterbird, the Eurasian coot *Fulica atra* . To this end, we sampled and genotyped nearly 300 coots at both innate (toll‐like receptors, TLRs) and adaptive (major histocompatibility complex, MHC) immune genes involved in pathogen recognition, while microsatellite genotypes were used as a reference for neutral differentiation. We found evidence of immunogenetic differentiation between coots from urban and non‐urban landscapes and identified particular immune gene variants and supertypes that differed in frequency between the two landscape types. However, the strongest immunogenetic differentiation was found among individual urban populations, which were also characterized by reduced immunogenetic diversity (allelic richness). Differentiation was higher at immune genes (TLR1LB and MHC class II) than neutral microsatellite loci, suggesting a contribution of selection. Overall, the observed patterns of immunogenetic differentiation likely reflect the combined effects of multiple processes, including independent urban colonization and associated founder effects, drift in spatially isolated urban populations, and selection promoting changes in the immune gene repertoire following each urban colonization event. Our results contribute to the understanding of molecular mechanisms underlying wildlife urbanization and suggest that immune genes, particularly those directly involved in pathogen recognition, may be at the forefront of adaptation to urban life. Our findings highlight the importance of considering immunogenetic variation in the conservation and management of wildlife populations in rapidly expanding urban landscapes.

## 1 Introduction

Urbanization has become one of the dominant forms of land transformation worldwide, driving the loss and fragmentation of natural habitats and ultimately leading to biodiversity declines and biotic homogenization (McKinney 2006, 2008). At the same time, urban environments create unique ecosystems that offer a multitude of benefits to at least some animal species, including relaxed pressure from natural predators, a milder microclimate, and access to various anthropogenic resources such as human‐derived food or artificial nesting sites (Shochat et al. 2006). However, these benefits may be effectively counterbalanced by the costs associated with urban life. Novel predators, different types of pollution (air, water, light, and noise), and anthropogenic disturbance or stress are commonly listed as factors that negatively affect the fitness of urban‐dwelling animals (Johnson and Munshi‐South 2017; Szulkin et al. 2020; Charmantier et al. 2024).

Urbanization may also be associated with substantial remodelling of pathogen and parasite community structure. Some pathogens are excluded from urban environments (e.g., due to the loss of natural hosts), whereas novel pathogens may emerge and colonize new hosts through interactions with humans or domestic animals (Hassell et al. 2017). Consequently, there is increasing evidence that colonization of urban environments requires broad immunological adaptation in wildlife (reviewed in Minias 2023), primarily in response to altered composition and richness of urban pathogen communities (Bradley and Altizer 2007). Although numerous studies have shown that urban environment can up‐ or downregulate host immune defence (Audet et al. 2016; Becker et al. 2019; Nwaogu et al. 2023), the signature of urbanization‐related immune adaptation is expected to be particularly pronounced in pathways involved in pathogen recognition, particularly in genes encoding molecules directly responsible for binding foreign (pathogen‐derived) antigens.

In vertebrates, foreign antigens can be effectively recognized by both arms of the immune system. Within the innate immune system, antigens are recognized by so‐called pattern recognition receptors, such as toll‐like receptors (TLRs). TLRs recognize evolutionarily conserved pathogen‐associated molecular patterns (PAMPs), such as lipopolysaccharide or flagellin, which are characteristic of a broad range of pathogens (Akira and Hemmi 2003). Consequently, TLR genes may show limited potential for rapid adaptation to dynamic changes in pathogen‐driven selective regimes across urbanization gradients (Minias 2023). In contrast, pathogen recognition within the adaptive immune system of jawed vertebrates relies on the major histocompatibility complex (MHC) molecules, which bind short peptides and present them to T cell receptors (TCRs). Presentation of a foreign antigen triggers T cell activation and initiates a cascade of immune responses aimed at pathogen elimination. There are two classes of MHC molecules primarily responsible for recognition of antigens from either intracellular (MHC class I, MHC‐I) or extracellular (MHC class II, MHC‐II) pathogens. Both classes are highly polymorphic, and balancing selection operating in natural vertebrate populations can maintain hundreds to thousands of MHC alleles (Dunn et al. 2013; Biedrzycka, O'Connor, et al. 2017; Biedrzycka, Sebastian, et al. 2017), which are often functionally specialized and exhibit narrow peptide‐binding repertoires (Kaufman 2018). Hence, MHC genes are known to respond to fine‐scale variation in pathogen‐driven selection (Spurgin and Richardson 2010) and are, therefore, expected to play a key role in immunogenetic adaptation to urban life (Minias 2023).

Despite these clear theoretical predictions, empirical evidence on whether and how urban colonization drives immunogenetic differentiation in animals remains limited. Convincing evidence for strong MHC differentiation between urban and non‐urban landscapes comes primarily from studies of canine mammals. In the endangered San Joaquin kit fox *Vulpes macrotis mutica* , only ~30% of MHC‐II alleles were shared between urban and non‐urban populations (Wilbert et al. 2020), while MHC‐linked microsatellite markers showed pronounced shifts in allele frequencies between urban and suburban/non‐urban populations of the red fox *Vulpes vulpes* and the coyote *Canis latrans* (DeCandia, Brzeski, et al. 2019; DeCandia, Henger, et al. 2019). In contrast, analyses of MHC variation in two European amphibian species, the common toad *Bufo bufo* and the smooth newt *Lissotriton vulgaris*, revealed no association between genetic differentiation and the level of urbanization at local scales (Babik et al. 2024). At the same time, information on urbanization‐related differentiation at innate pathogen‐recognition genes is virtually lacking. Taking all this into account, it remains unclear whether immunogenetic differentiation across urbanization gradients is taxon‐ and locus‐specific, and substantially greater research effort—spanning phylogenetically divergent taxa and multiple immune markers—is required to draw robust general conclusions.

In this study, we aimed to test whether colonization of urban environment drives immunogenetic differentiation in a common waterbird, the Eurasian coot *Fulica atra* . To this end, we sampled four pairs of urban and non‐urban coot populations across Poland and conducted extensive genotyping of both innate (TLRs) and adaptive (MHC) pathogen‐recognition genes. We also used microsatellite genotypes to quantify neutral genetic differentiation arising from demographic processes. Previous molecular research on coots provided support for independent urban colonization, with each city apparently colonized through separate immigration events of birds from neighbouring non‐urban source populations (Minias et al. 2017). Preliminary analyses of MHC‐II variation showed significant differentiation between two urban and two non‐urban populations, suggesting potential immunogenetic adaptation (Pikus et al. 2021). However, it remains unresolved whether this urbanization‐related immunogenetic differentiation follows a general pattern across replicated pairs of urban and non‐urban populations. It also remains unclear whether independent urban colonization history, spatial isolation of urban habitats, and population‐specific pathogen‐driven selection would promote strong immunogenetic differentiation among individual urban populations. Finally, it is unknown whether innate and adaptive immune genes involved in pathogen recognition show a similar level of differentiation among landscapes and populations. Here, we hypothesized that independent urban colonization may produce a complex pattern of immunogenetic differentiation characterized by both general and local effects. Specifically, we predicted significant immunogenetic differentiation between urban and non‐urban landscapes, but also significant differentiation between independently established and spatially isolated urban populations. We also predicted that more evolutionarily conserved innate immune genes should be less responsive to dynamic changes in selective pressure across landscapes and, thus, show weaker differentiation among urban and non‐urban populations compared to the hyper‐polymorphic MHC genes.

## 2 Methods

### 2.1 Study Sites and Sample Collection

Sampling took place in four pairs of urban and non‐urban populations of the Eurasian coot in Poland. Urban sampling sites were situated in four major cities: Łódź (51.8019° N, 19.4726° E), Katowice (50.3009° N, 18.9922° E), Poznań (52.4276° N, 16.8772° E), and Warszawa (52.2818° N, 20.9850° E) (Figure 1). All four agglomerations are among the most urbanized areas in the country and are characterized by large human populations (over half a million inhabitants each). Urban coots were sampled either at artificial reservoirs surrounded by dense urban development or in urban parks subject to high anthropogenic pressure. All urban sampling sites were also characterized by highly transformed habitats, including concrete embankment and little or no reed vegetation. The distance among urban sampling sites ranged from ca. 120–260 km. Each urban population was paired with a non‐urban population located 20–40 km away. Non‐urban sampling sites were situated on natural and semi‐natural waterbodies (mostly lakes and fishponds) characterized by limited anthropogenic pressure (e.g., through restricted public access at commercial fishponds). All non‐urban sites were mostly surrounded by agricultural areas or wetlands and provided natural‐like habitats for coots, including extensive patches of reed vegetation.

**FIGURE 1:**
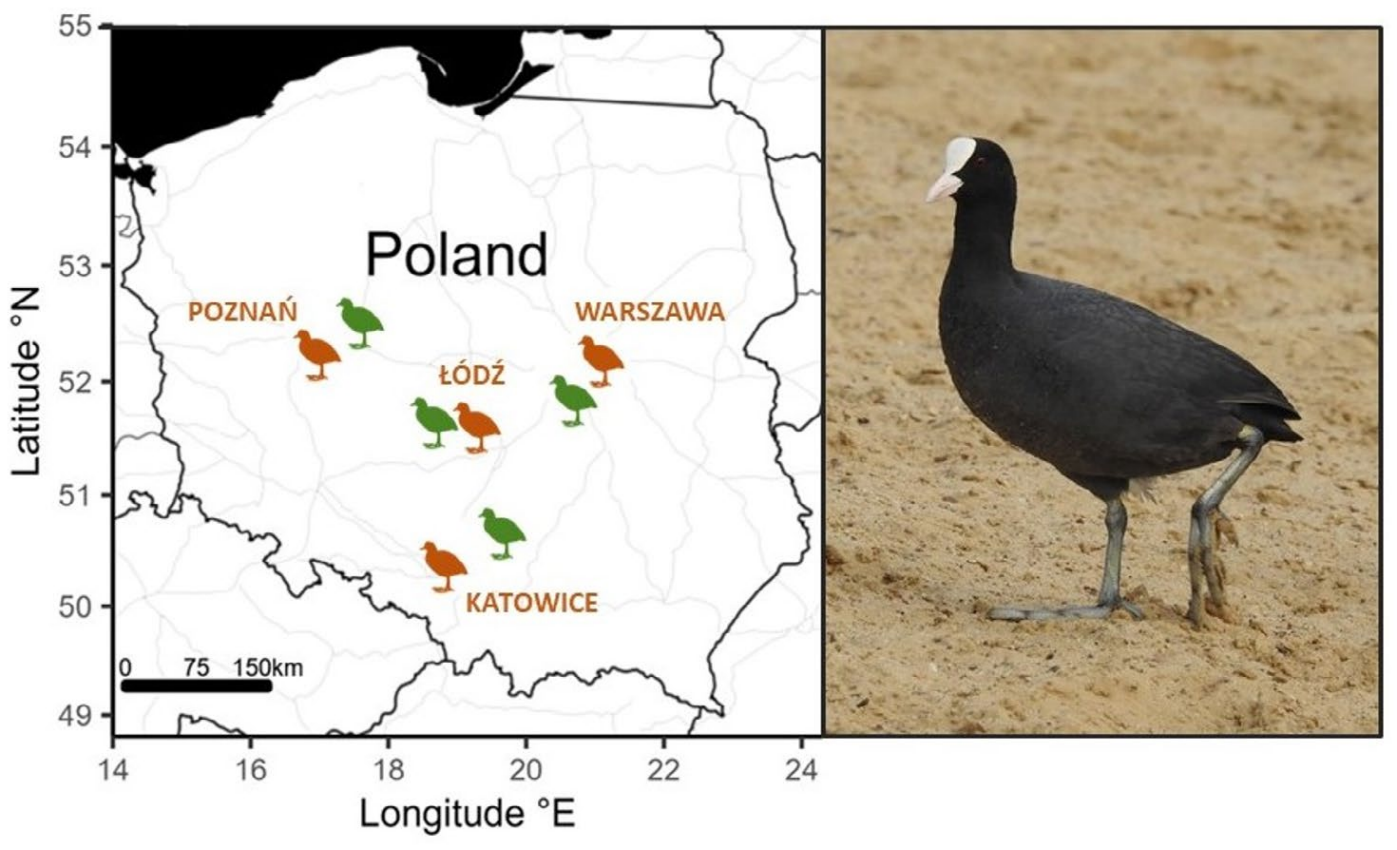
Locations of four pairs of urban (orange) and non‐urban (green) Eurasian coot populations sampled in Poland.

Fieldwork was conducted from 2016 to 2023. Adult coots were captured during the breeding season (March–July) using nest traps made of braided fishing line or by hand (the latter exclusively at urban sampling sites). In total, we captured 294 coots across all urban and non‐urban populations (30–48 individuals per site), but we aimed to genotype ~30 individuals per each genetic marker per population. Each captured coot was marked with a metal ring on the left tarsus and a plastic neckband with an alphanumeric code to facilitate individual identification in the field and avoid unintentional recaptures. Following capture, we collected approx. 50 μL of blood by puncturing the tarsal vein with a sterile needle. Blood was collected into Eppendorf tubes with 96% ethanol and stored at 4°C until analysis.

### 2.2 Immunogenetic Variation

Genomic DNA was extracted using a DNA Purification Kit (Euryx, Gdańsk, Poland) following the manufacturer's protocol. Extracted DNA was used to genotype both innate (TLRs) and adaptive (MHC) immune genes in coots.

#### 2.2.1 Toll‐Like Receptors

We genotyped four TLR loci that encode receptors binding different classes of pathogen‐derived ligands. Our targeted loci included TLR1LB, TLR4, and TLR5, which detect diacylated lipoproteins, lipopolysaccharide, and flagellin of bacterial and protozoan origin, and TLR3, which recognizes double‐stranded viral RNA (Alcaide and Edwards 2011). We amplified these loci using primers designed by Alcaide and Edwards (2011) that target conserved coding regions of avian TLR genes. Specifically, all primer pairs amplify segments of the extracellular domains enriched in leucine‐rich repeats (LRRs), which mediate ligand binding and the recognition of pathogen‐associated molecular patterns (PAMPs). Highly conserved motifs such as the cytoplasmic Toll/interleukin 1 receptor/resistance domain were intentionally not targeted by the primers. PCR amplifications followed original protocols provided by Alcaide and Edwards (2011) and amplicons were sequenced bidirectionally using the Sanger method. High quality sequences were obtained for an average of 25.3 ± 4.9 [SD] individuals per population per locus (Table 1). The sequences were aligned and trimmed to equal lengths (from 849 to 1092 bp per locus, Table 1) using Geneious v10.0.5 (Biomatters Ltd., Auckland, New Zealand). Comparisons with annotated TLR sequences from the domestic chicken *Gallus gallus* (Temperley et al. 2008) indicated that approx. 80% of codons in our coot TLR sequences corresponded to LRR regions across all four loci. Raw (unphased) consensus TLR sequences were phased into haplotypes using the PHASE algorithm (Stephens and Donnelly 2003), with a burn‐in of 1000 iterations, 1000 final iterations and a thinning interval of 10, as implemented in DnaSP v6.10.03 (Rozas et al. 2017). Phasing was performed separately for each TLR locus using the complete across‐population sequence alignments. Phasing reliability was enhanced by the relatively high frequency of homozygous genotypes and genotypes heterozygous at only a single segregating site (on average 59.3% across loci).

**TABLE 1:** Polymorphism of innate (TLR) and adaptive (MHC) immune genes in Eurasian coots from four pairs of urban and non‐urban populations.

Immunity	Gene	Coding region	Length (bp)	Sample size (no. individuals)	*N*_A_	Sequence polymorphism		
						S	*η*	*π*
Innate	TLR1LB	Exon 1	1038	224	78 (39)	39	40	0.0025
	TLR3	Exon 4	1031	151	40 (18)	29	29	0.0018
	TLR4	Exon 3	849	228	11 (6)	10	10	0.0005
	TLR5	Exon 1	1098	207	29 (16)	17	17	0.0033
Adaptive	MHC‐I	Exon 3	263	244	983 (773)	244	474	0.1385
	MHC‐II	Exon 2	270	225	164 (158)	102	141	0.1150

Abbreviations: *η*, total number of mutations; *π*, nucleotide diversity; *N*_A_, number of allelic variants (number of unique amino acid variants reported in parentheses); S, number of segregating sites.

#### 2.2.2 Major Histocompatibility Complex

We genotyped both MHC‐I and MHC‐II in all four population pairs. Genotyping focused on MHC‐I exon 3 and MHC‐IIβ exon 2, which encode the peptide‐binding regions directly involved in antigen presentation. A recent analysis of the Eurasian coot genome revealed 32 contigs containing full‐length MHC‐I genes (Pikus and Minias 2022), suggesting that the primers originally used to amplify MHC‐I in coots (*MHCI‐int2F* and *MHCI‐ex4R*) may not have captured the full extent of MHC‐I variation in this species (max. 10 MHC‐I alleles detected per individual; Pikus et al. 2022). Therefore, we redesigned the primers based on the available genomic resources, yielding a new primer pair *Fuat‐MHCI‐Ex3Fw* (5′‐CCGTCTCCCTGGTCGTGT‐3′) and *Fuat‐MHCI‐Ex3Rv* (5′‐YYTSRYCWTTCCTCTYYAGC‐3′). These primers anneal to a conserved region of intron 2 adjacent to exon 3 and a relatively conserved junction between exon 3 and intron 3, respectively. The primers amplified 263 bp of MHC‐I exon 3 (out of a total exon length of 276 bp). For MHC‐II genotyping, we used *Fuat‐Ex2Fw* and *Fuat‐Ex2Rv* primers designed for the Eurasian coot by Alcaide et al. (2014), which anneal to conserved intron regions flanking MHC‐IIβ exon 2 and amplify the entire exon length (270 bp). All PCR amplifications were conducted using fusion primers containing Illumina Nextera Transposase adapter sequences (Illumina Corp., San Diego, CA, USA) and 7‐bp barcodes for amplicon identification. The PCR amplification protocols followed Alcaide et al. (2014) with the number of cycles limited to 25 to suppress chimera formation. Library preparation followed Pikus and Minias (2022), and 2 × 300 bp Illumina MiSeq platform was used for library sequencing.

Raw paired‐end Illumina reads were merged using the BBMerge algorithm implemented in Geneious v10.0.5. All further processing (de‐multiplexing, clustering and filtering) of merged reads was conducted using a stand‐alone version of AmpliSAS software (Sebastian et al. 2016, 2024). In the clustering step, which aims to identify reads resulting from genotyping errors and cluster them with reads identified as true alleles, we set 1% substitution error rate, 0.001% indel error rate and 25% minimum dominant frequency threshold, following recommendations by Biedrzycka, O'Connor, et al. (2017) and Biedrzycka, Sebastian, et al. (2017). In the filtering step, we removed clusters representing putative chimeras and other low‐frequency genotyping artefacts. We used a lower minimum per amplicon frequency threshold for MHC‐I (0.8%) than MHC‐II (3%), as we expected to detect substantially more alleles per amplicon at MHC‐I (> 30 loci suggested by genome analysis; Pikus and Minias 2022) than at MHC‐II (three loci; Alcaide et al. 2014). For the same reason, the maximum amplicon depth used for the processing was set to 10,000 reads for MHC‐I and 5000 reads for MHC‐II. The minimum depth of true alleles was set to 4000 and 2000 reads for MHC‐I and MHC‐II, respectively. The average depth of true alleles was 7536 ± 59 [SE] reads for MHC‐I and 4313 ± 28 [SE] reads for MHC‐II. Allele reproducibility was 92% (MHC‐I) and 99% (MHC‐II), as assessed using 22 and 20 technical replicates, respectively, where each replicate was obtained through independent PCR amplification and sequencing of the same sample. Final genotype data were obtained for 244 (MHC‐I) and 225 (MHC‐II) individuals. All sequences were aligned and trimmed to exonic regions in Geneious v10.0.5. At MHC‐I we recorded up to six sequences with stop codons per amplicon, suggesting that our primers targeted up to three MHC‐I pseudogenes. All such sequences were filtered out prior to final analysis. At MHC‐II we detected no stop codons or frame‐shift mutations in the sequences, indicating that all sequences represented putatively functional MHC allelic variants.

As similar MHC allelic variants may have similar properties in terms of antigen binding, coarser representation of functional MHC variation may be more relevant to investigate population‐level responses to pathogen‐driven selection (Biedrzycka et al. 2018). To this end, we clustered MHC‐I and MHC‐II allelic variants into functional supertypes, i.e., groups of alleles showing similar functional (binding) properties. The clustering was based on the positively selected sites detected previously at the MHC genes in the Eurasian coot (15 MHC‐I and 24 MHC‐II sites; Pikus and Minias 2022), which likely correspond to peptide‐binding residues. We first used the *DistCalc* function from the *MHCtools* package (Roved et al. 2022) developed for the R statistical environment (R Foundation for Statistical Computing, Vienna, Austria) to generate matrices of physicochemical (Sandberg) distances between alleles. Then, the matrices were used as input for k‐means clustering and discriminant analysis of principal components (DAPC) implemented in the *adegenet* R package (Jombart 2008). The number of clusters was selected based on the minimum value of Bayesian information criterion (BIC) for MHC‐II or location of the inflection point of the BIC curve for MHC‐I (no clear minimum BIC value). The number of principal components (PCs) in the DAPC analysis was selected based on the maximum α‐score (identified with the *optim.a.score* function). The entire procedure was repeated 1000 times and we used the average posterior probabilities to derive a consensus assignment of alleles to clusters (supertypes).

### 2.3 Neutral Genetic Variation

To quantify neutral genetic differentiation between urban and non‐urban coot populations, we used a panel of 15 non‐genic microsatellite markers, including ten markers originally designed for the Eurasian coot (Lv et al. 2017). The remaining five markers were originally developed for other rallid species, including the black rail *Laterallus jamaicensis* (B106) (Molecular Ecology Resources Primer Development Consortium 2009), the king rail *Rallus elegans* (KiRa9, KiRa10 and KiRa16; Brackett et al. 2013) and the Tasmanian native‐hen *Tribonyx mortierii* (Tm27; Buchan 2000). All these markers were previously reported to successfully cross‐amplify in the Eurasian coot and showed no linkage disequilibrium (Chyb et al. 2023). Here, microsatellite markers were amplified in multiplexes (3–4 markers per multiplex) using forward primers fluorescently labelled with 6‐FAM, VIC, ROX, and HEX and all amplifications were conducted following the protocols described in Chyb et al. (2023). Fragment size analysis was conducted using ABI 3730XL capillary sequencer (Applied Biosystems, Foster City, CA, USA) and allele sizes were scored against GeneScan 600 LIZ Standard (Applied Biosystems) in Geneious v.10.0.5. We obtained microsatellite genotypes for 246 individuals (27–33 per population). Repeatability of allele scoring between two independent genotyping runs was high (98.3%), as assessed with independent re‐genotyping of 120 single‐marker amplicons. We found no evidence of genotyping errors due to stuttering or large allele dropout and the estimated frequency of null alleles was low (mean 0.016 ± 0.035 [SD] across all markers; Table S1), as assessed using Micro‐Checker 2.2.3 (van Oosterhout et al. 2004). The majority (94.2%) of population‐specific tests (*χ* ^2^ with Bonferroni correction) revealed no significant deviation of allele frequencies from Hardy–Weinberg equilibrium (HWE), as assessed using GenAlEx v.6.51b2 (Peakall and Smouse 2012). The number of alleles varied from 7 to 54 (mean 19.2 ± 12.7 [SD]) and observed heterozygosity ranged from 0.51 to 0.94 per marker (mean 0.75 ± 0.15 [SD]; Table S1).

### 2.4 Immunogenetic Diversity

Sequence polymorphism of immune genes (TLRs and MHC) was quantified as the number of segregating sites (S), the total number of mutations (*η*), and nucleotide diversity (*π*) across all detected allelic variants using DnaSP v6.10.03. Allelic richness at TLRs (locus‐specific and across loci), MHC‐I, MHC‐II, and microsatellites was compared between urban and non‐urban populations using the *hierfstat* R package (Goudet 2005). The significance of differences in allelic richness was determined using permutation tests (population labels permutated among individuals, 1000 permutations), and the standardized effect size (SES) was calculated as (T_obs_—*μ*_perm_)/*σ*_perm_, where T_obs_ is the observed difference in mean allelic richness, while *μ*_perm_ and *σ*_perm_ are the mean and standard deviation of the permutation null distribution, respectively.

### 2.5 Population Differentiation

To quantify genetic differentiation between landscapes and among populations, we used Jost's D, which measures allelic differentiation in terms of the effective number of alleles and, thus, can be calculated for multi‐locus data, such as the MHC, for which true heterozygosity remains unknown (Jost 2008). Jost's D values for neutral genetic markers (microsatellites) and TLR genes were calculated using the *strataG* R package (Archer et al. 2017). TLR differentiation was quantified both separately for each locus and across all four loci. Multi‐locus MHC data were transformed into an allele presence‐absence matrix (as in a dominant marker) and Jost's D values were calculated using the *SpadeR* R package (Chao et al. 2016). The significance of all D values was tested using 1000 permutations. For immune genes, we first quantified differentiation using allelic variants, but to better capture functional immune variation, we also re‐ran analyses using amino acid variants for TLRs and functional supertypes for MHC.

We used generalized linear models (GLMs) to test for differences in pairwise population differentiation (Jost's D) among three types of comparisons: (i) between urban and non‐urban landscape (i.e., among populations from different landscapes), (ii) within the urban landscape (i.e., among different urban populations), and (iii) within the non‐urban landscape (i.e., among different non‐urban populations). These tests were run for both immune genes and neutral microsatellite markers. GLMs were also used to test whether landscape type was associated with the presence/absences of particular TLR amino acid variants (separately for each locus) and MHC functional supertypes (separately for each class). In these models, landscape type was used as the response variable, while predictors included presence/absence of amino acid variants or supertypes occurring at 10%–90% frequency. The models were fitted with a binomial error distribution. Finally, we tested for differences in pairwise population differentiation among different marker types. Here, we used general linear mixed models (GLMMs) with Josts's D used as the response variable, marker type as a fixed factor, and the identity of each population pair as a random factor. All linear models were run in the *lme4* R package (Bates et al. 2015). Effect sizes for individual predictors were quantified using pseudo‐R^2^ values in the *performance* R package (Lüdecke et al. 2021). All values are reported as means ± SE.

## 3 Results

### 3.1 Immunogenetic Polymorphism

Four TLR loci showed varying levels of polymorphism: TLR1LB had the highest number of alleles detected (N_A_ = 78), but nucleotide diversity was highest at TLR5 (*π* = 0.0033) (Table 1). TLR4 showed the lowest polymorphism in terms of both the number of alleles detected (N_A_ = 11) and nucleotide diversity (π = 0.0005) (Table 1). The number of unique amino acid variants varied between 6 and 39 among loci (Table 1).

We detected a maximum of 51 MHC‐I and 6 MHC‐II alleles per individual, consistent with the amplification of at least 26 and 3 loci, respectively (assuming heterozygosity at each locus). The mean number of MHC alleles per individual was 36.14 ± 0.34 for MHC‐I and 2.93 ± 0.06 for MHC‐II. MHC‐I was more polymorphic than MHC‐II, both in terms of the total number of detected alleles (983 vs. 164) and nucleotide diversity (0.1385 vs. 0.1150) (Table 1). MHC‐I and MHC‐II alleles were clustered into 20 and 10 functional supertypes, respectively, comprising 6–159 alleles per supertype for MHC‐I and 5–28 alleles per supertype for MHC‐II.

### 3.2 Genetic Diversity of Urban and Non‐Urban Populations

We found evidence of significantly lower TLR allelic richness (across all loci) in urban than non‐urban populations (21.90 vs. 25.63 alleles per locus, respectively; permutation test: *p* = 0.020, SES = 2.36; Figure S1). However, locus‐specific permutation tests revealed significant differences at only one (TLR1LB) locus (*p* = 0.041, SES = 2.07), but not at the other loci (all *p* > 0.05). A large proportion (60.5%) of all TLR alleles was private to either landscape, with a higher number of private alleles in non‐urban than urban coot populations (56 vs. 39 alleles across all loci).

Similarly to TLRs, MHC‐I and MHC‐II showed significantly higher allelic richness in non‐urban than urban populations (permutation tests: *p* < 0.001 and SES = 3.81 for MHC‐I; *p* = 0.050 and SES = 1.95 for MHC‐II; Figure S2). In total, 52.0% of MHC‐I alleles and 55.5% of MHC‐II alleles were private to either landscape, with a higher number of private alleles detected in non‐urban than urban populations (300 vs. 211 alleles for MHC‐I and 52 vs. 39 alleles for MHC‐II). There was also a non‐significant tendency towards lower microsatellite diversity in urban populations (permutation tests: *p* = 0.069, SES = 1.79). Notably, the effect size for the observed reduction in genetic diversity in urban populations was over twice as large for MHC‐I as for microsatellites.

### 3.3 TLR Differentiation Between and Within Landscapes

We detected several cases of significant pairwise population differentiation across all TLR loci, but these were only apparent for urban/urban and urban/non‐urban comparisons, while no significant differentiation was found among non‐urban populations (Table 2). Significant locus‐specific differentiation was also mainly observed for urban/urban and urban/non‐urban population comparisons (Tables S2–S5). Consistently, we found significantly stronger multi‐locus TLR allelic differentiation among urban populations than among non‐urban populations (D = 0.025 ± 0.007 vs. D = 0.002 ± 0.003; *β* = 0.023 ± 0.007, *p* = 0.007; Table S6; Figure 2A). Differentiation among urban and non‐urban populations showed intermediate values (Figure 2A). Analysis of multi‐locus TLR amino acid differentiation showed a similar, although only marginally significant pattern (Table S7). Locus‐specific analyses showed consistent patterns of differentiation for TLR3 allelic and TLR4 amino acid variants, with both analyses indicating significantly stronger differentiation among urban than among non‐urban populations and intermediate differentiation among urban and non‐urban populations (Tables S6 and S7; Figure 2B,C).

**TABLE 2:** Differentiation at TLR genes (across all four loci) among landscapes and populations in the Eurasian coot.

Landscape/population		Urban				Non‐urban			
		Katowice	Łódź	Poznań	Warszawa	Katowice	Łódź	Poznań	Warszawa
Urban	Katowice	—	0.008	0.012	**0.035**	0.007	−0.005	0.000	−0.004
	Łódź	0.009	—	0.016	0.016	0.014	0.018	0.017	−0.004
	Poznań	0.018	**0.031**	—	**0.041**	0.001	0.001	**0.027**	0.003
	Warszawa	0.017	0.015	**0.058**	—	**0.029**	**0.036**	**0.039**	**0.027**
Non‐urban	Katowice	0.002	0.013	0.007	**0.030**	—	0.003	0.018	0.011
	Łódź	−0.002	0.023	0.011	**0.039**	0.006	—	0.009	−0.005
	Poznań	−0.008	0.013	0.016	0.011	0.004	−0.001	—	0.006
	Warszawa	−0.010	0.008	0.000	0.022	0.014	−0.003	−0.009	—

*Note:* Differentiation was quantified using Jost's D based on allelic variants (below the diagonal) and amino acid variants (above the diagonal). Significant values are marked in bold.

**FIGURE 2:**
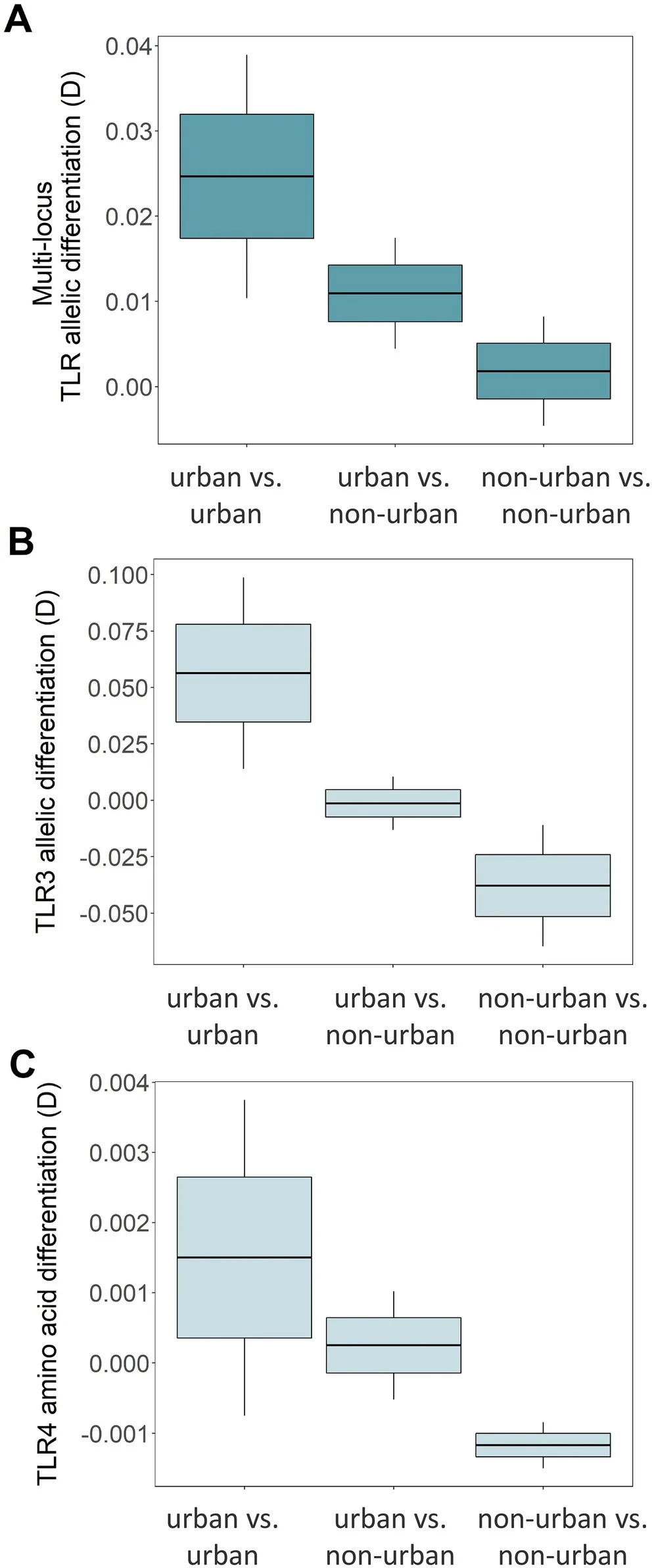
Population differentiation at TLR allelic variants across all four loci (A), TLR3 allelic variants (B) and TLR4 amino acid variants (C) among urban populations, between urban and non‐urban populations, and among non‐urban populations in the Eurasian coot. Central line—mean, box—SE, whiskers—95% confidence interval.

The analysis of particular amino acid variants revealed a significant association of only one TLR1LB variant (aa_TLR1LB*02) with landscape type, showing larger frequency in urban than non‐urban coot populations (Table S8; Figure 3A). However, the effect size of this association was low (pseudo‐*R* ^2^ = 0.021). No significant between‐landscape differences in the frequencies of particular amino acid variants were found for the other TLR loci (Table S8).

**FIGURE 3:**
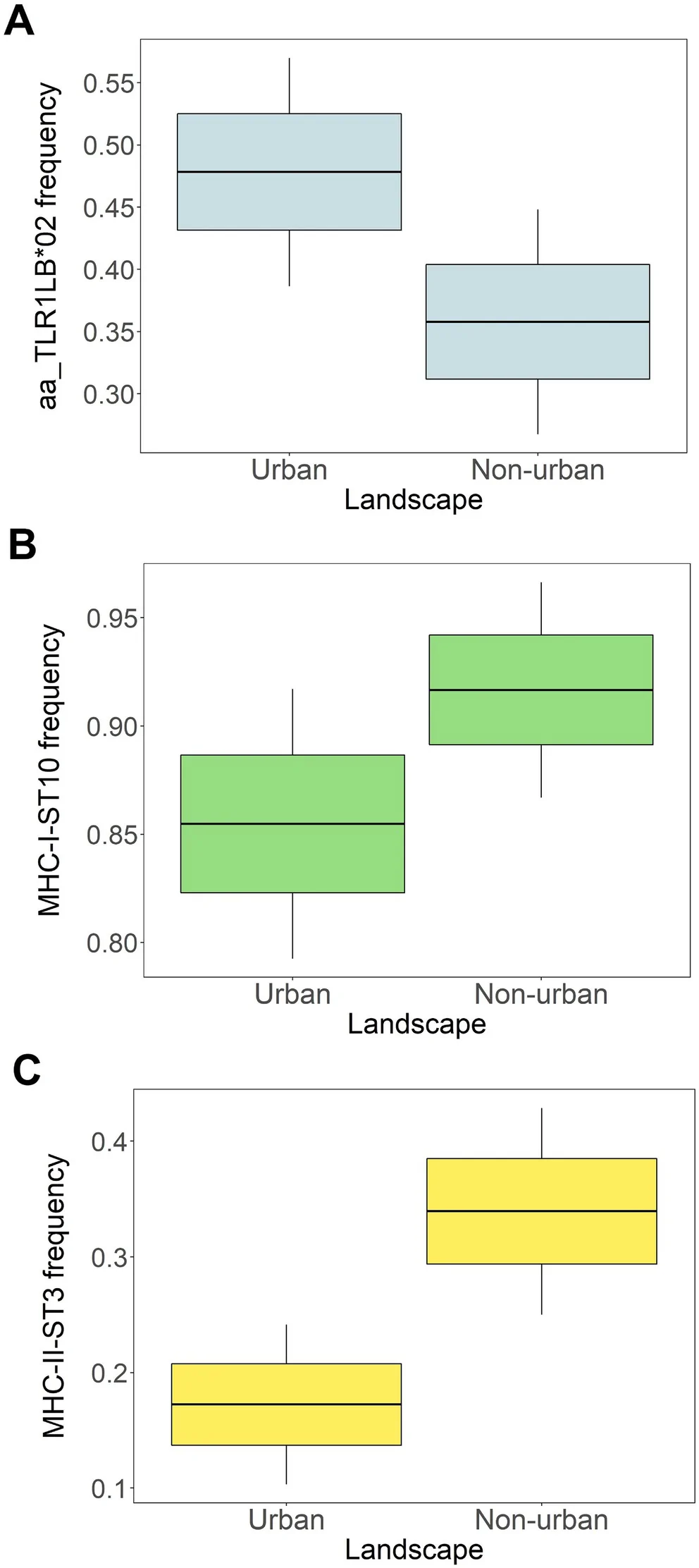
Differences in the frequency of aa_TLR1LB*02 amino acid variant (A), MHC‐I‐ST10 functional supertype (B) and MHC‐II‐ST3 functional supertype (C) between urban and non‐urban landscape in the Eurasian coot. Central line—mean, box—SE, whiskers—95% confidence interval.

### 3.4 MHC Differentiation Between and Within Landscapes

Similarly to TLRs, significant MHC‐I and MHC‐II differentiation was detected exclusively for pairwise comparisons of urban/urban and urban/non‐urban populations (Table 3). Consistently, we found significantly stronger MHC‐I differentiation among urban populations than among non‐urban populations (*β* = 0.067 ± 0.021; *p* = 0.005) and a similar pattern was found for MHC‐II (*β* = 0.279 ± 0.066; *p* < 0.001) (Table S9, Figure 4). In both cases, these differences yielded moderate‐to‐high effect sizes (*R* ^2^ = 0.28 and *R* ^2^ = 0.41, respectively). For both MHC‐I and MHC‐II, differentiation between urban and non‐urban populations showed intermediate levels (Figure 4). No significant differentiation in overall MHC functional supertype composition was found among any pairs of populations (all *p* > 0.05).

**TABLE 3:** Differentiation at MHC genes among landscapes and populations in the Eurasian coot.

Landscape/population		Urban				Non‐urban			
		Katowice	Łódź	Poznań	Warszawa	Katowice	Łódź	Poznań	Warszawa
Urban	Katowice	—	**0.391**	0.159	**0.291**	0.000	0.180	0.097	0.196
	Łódź	0.021	—	**0.268**	**0.552**	0.175	**0.264**	**0.269**	0.093
	Poznań	0.032	0.031	—	**0.253**	0.000	0.108	0.000	0.000
	Warszawa	**0.125**	**0.107**	**0.083**	—	**0.243**	**0.360**	**0.271**	**0.338**
Non‐urban	Katowice	0.003	0.000	0.000	**0.098**	—	0.000	0.000	0.018
	Łódź	0.013	0.016	0.000	**0.109**	0.000	—	0.087	0.098
	Poznań	0.000	0.012	0.000	**0.077**	0.000	0.000	—	0.038
	Warszawa	0.000	0.038	0.005	**0.094**	0.000	0.000	0.000	—

*Note:* Differentiation was quantified using Jost's D based on MHC‐I (below the diagonal) and MHC‐II (above the diagonal) allelic variants. Significant values are marked in bold.

**FIGURE 4:**
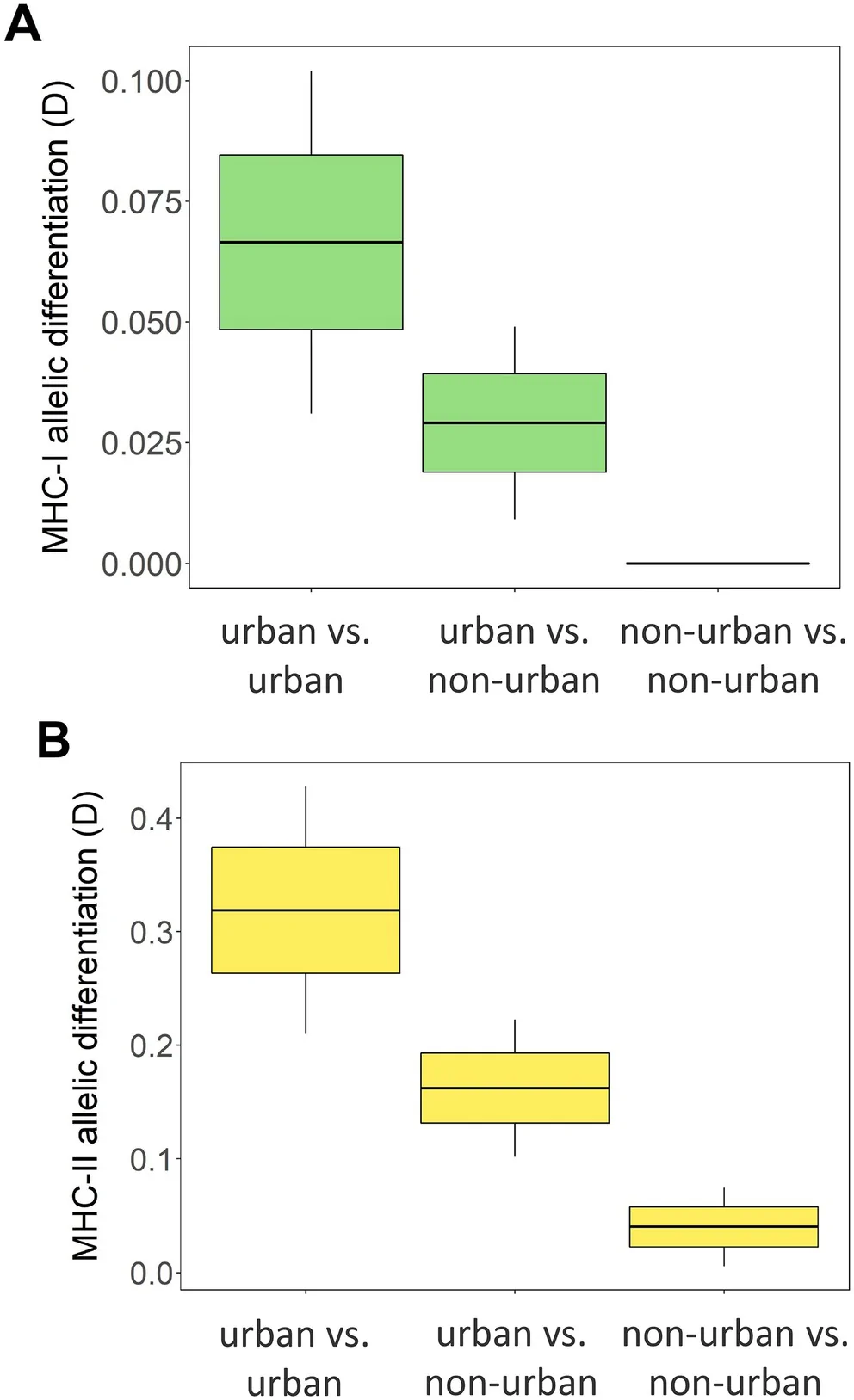
Population differentiation at MHC‐I (A) and MHC‐II (B) allelic variants among urban populations, between urban and non‐urban populations, and among non‐urban populations in the Eurasian coot. Central line—mean, box—SE, whiskers—95% confidence interval.

We detected significant associations of landscape type with one MHC‐I and one MHC‐II functional supertype (ST10 and ST3, respectively; Table S10). Both supertypes were significantly less frequent in urban than non‐urban landscapes (Figure 3B,C). The effect sizes of these associations were relatively low (pseudo‐*R* ^2^ = 0.019 and 0.039, respectively).

### 3.5 Immunogenetic Versus Neutral Genetic Differentiation

Neutral microsatellite loci showed low, but significant differentiation between landscapes (D = 0.007, *p* = 0.007). Consistent with patterns observed for immunogenetic markers, significant pairwise population differentiation was restricted to urban/urban and urban/non‐urban comparisons (Table S11), and differentiation among urban populations was significantly stronger than among non‐urban populations (Table S12, Figure S3). Overall population differentiation at MHC‐II and TLR1LB (but not at MHC‐I and other TLR loci) was significantly stronger than differentiation at neutral microsatellite loci (MHC‐II: *β* = 0.138 ± 0.020, *p* < 0.001; TLR1LB: *β* = 0.113 ± 0.020, *p* < 0.001; Table S13, Figure 5). The effect sizes of these significant differences were moderate (pseudo‐*R* ^2^ = 0.165 and 0.116, respectively). Stronger differentiation at MHC‐II and TLR1LB compared to microsatellites retained significance when the analysis was restricted to: (i) comparisons among urban and non‐urban populations (MHC‐II: *β* = 0.131 ± 0.025, *p* < 0.001; TLR1LB: *β* = 0.105 ± 0.025, *p* < 0.001) and (ii) comparisons among urban populations (MHC‐II: *β* = 0.255 ± 0.046, *p* < 0.001; TLR1LB: *β* = 0.135 ± 0.046, *p* = 0.006). The effect size was particularly high for the difference between MHC‐II and microsatellite differentiation among urban populations (pseudo‐*R* ^2^ = 0.433).

**FIGURE 5:**
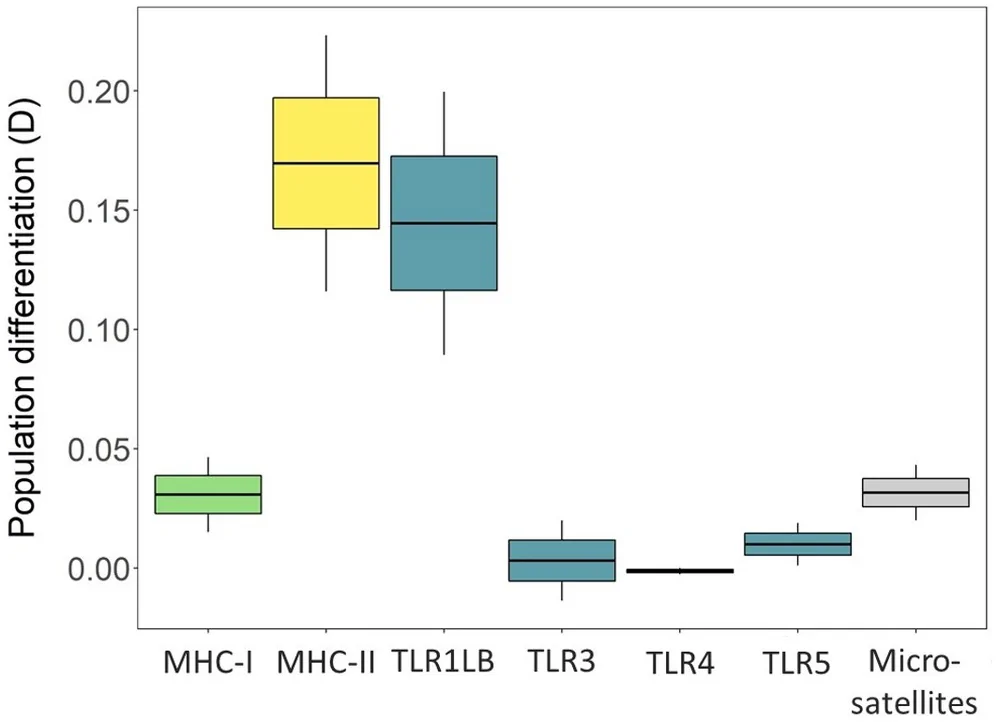
Comparison of population differentiation across immunogenetic (MHC and TLR) and neutral genetic (microsatellites) markers in the Eurasian coot. Central line—mean, box—SE, whiskers—95% confidence interval.

## 4 Discussion

In this study, we found evidence that colonization of isolated urban habitat patches (i.e., cities) was associated with considerable immunogenetic differentiation in the Eurasian coot. First, we found several cases of significant differentiation at TLR and MHC genes between urban and non‐urban populations. We also found significant differences in the frequencies of particular TLR/MHC variants/supertypes between urban and non‐urban landscapes. Urban populations were characterized by reduced immunogenetic diversity (lower allelic richness), suggesting that some allelic variants may have been disfavoured by selection or lost through neutral processes in urban environments. At the same time, the strongest immunogenetic differentiation was detected among individual urban populations. This pattern may reflect independent urban colonization processes, whereby each city was colonized through separate immigration of birds from nearby non‐urban source populations. However, it may also have been reinforced by local adaptation to the environmental and ecological characteristics of individual urban sites, likely enhanced by limited gene flow among spatially isolated urban populations. Indeed, population differentiation at some immune loci (MHC‐II and TLR1LB) was significantly greater than differentiation at neutral microsatellite markers. This suggests that the observed level of immunogenetic differentiation, at least for some genes, is unlikely to be fully explained by demographic history and drift, but may instead reflect a contribution of pathogen‐driven selection. Given the current lack of empirical data on landscape‐related variation in pathogen communities of coots, we suggest that incorporating information on pathogen pressure into future research may provide more mechanistic insights into the immunogenetic differentiation between urban and non‐urban coot populations.

There is accumulating evidence that urbanization leaves a genetic footprint in animal populations (Johnson and Munshi‐South 2017). Although genome‐wide association studies of non‐model organisms in the context of urbanization are still rare, they have consistently revealed broad genetic changes associated with urban colonization. While genes related to neural function, behaviour, cognition, metabolism, and hormonal responses have often been among the strongest candidates for genetic adaptation to urban life (Perrier et al. 2018; Mueller et al. 2020; Salmón et al. 2021), some studies have also detected enrichment of genes directly related to immune function. For instance, genomic research in the white‐footed mouse *Peromyscus leucopus* identified a number of genes associated with innate and adaptive immunity (including genes involved in TLR and TCR signalling pathways) that experienced divergent selection between urban and non‐urban populations (Harris et al. 2013; Harris and Munshi‐South 2017). Genes involved in the TLR signalling pathway (along with several other immune genes) were also enriched in comparisons between giant toads *Rhinella horribilis* from natural and human‐modified landscapes (Soria‐Ortiz and Vázquez‐Domínguez 2026). Among birds, the study on the burrowing owl *Athene cunicularia* revealed several immune genes located in genomic windows with high urban‐specific sweep probability (Mueller et al. 2020). Consistently, our targeted sequencing of candidate immune genes involved in pathogen recognition provided support for immunogenetic differentiation between urban and non‐urban populations of the Eurasian coot. This immunogenetic differentiation between landscapes was also detectable at the level of individual sequence variants or functional supertypes. Specifically, we detected MHC‐I and MHC‐II supertypes that showed reduced frequency in the urban landscape, while one TLR1LB amino acid variant occurred at a higher frequency in urban populations. We suggest that this differentiation may reflect (at least in part) immunogenetic adaptation to the urban environment, where different variants/supertypes may be favoured by pathogen‐mediated selection in different types of landscape.

Although we lacked data on fitness across our study populations, the suggested adaptive scenario was indirectly supported by substantially stronger differentiation at MHC‐II and TLR1LB compared to differentiation at neutral microsatellite markers. This suggests that demographic history and genetic drift are unlikely to fully explain the observed patterns of immunogenetic variation across populations and landscapes. Highly polymorphic MHC genes are known to respond dynamically to spatial and temporal changes in pathogen pressure through fluctuating selection, which can promote adaptation to local ecological and environmental conditions (Cortazar‐Chinarro et al. 2025). Consistently, there is a large body of evidence for pathogen‐mediated local adaptation at MHC genes (e.g., Eizaguirre et al. 2012; Kyle et al. 2014), and urbanization‐related changes in MHC allelic composition have previously been reported for several species, mostly mammals (DeCandia, Brzeski, et al. 2019; DeCandia, Henger, et al. 2019; Wilbert et al. 2020). Our results indicate that similar urbanization‐related processes may also occur at less polymorphic TLR genes. As TLRs recognize evolutionarily conserved pathogen‐associated molecular patterns, they are often expected to be less responsive to dynamic changes or fluctuations in selective pressures (Minias and Vinkler 2022). However, despite strong evolutionary constraints, some evidence for short‐term positive selection on TLRs is accumulating. A strong decline in the frequency of a minor TLR3 allele over just a few generations in the Seychelles warbler *Acrocephalus sechellensis* provides a notable example (Davies et al. 2021). Our results suggest that TLR genes may also be involved in rapid local (urbanization‐related) adaptation, although more research is required to provide robust empirical support for this scenario. So far, the expression of avian TLR genes has been linked to urbanization and urban colonization history (Capilla‐Lasheras et al. 2017; Kilvitis et al. 2019), although it remains unclear whether these observations reflect phenotypic plasticity or adaptive processes.

Apart from immunogenetic differentiation among landscapes, we also recorded reduced TLR and MHC diversity in urban populations. In general, reduced genetic diversity in urban populations is among the most commonly reported urban evolutionary patterns, primarily attributed to founder effects and resulting genetic drift (Johnson and Munshi‐South 2017). However, urbanization‐related loss of genetic diversity is particularly strong in species with limited dispersal capabilities, such as amphibians or small mammals (e.g., Noël et al. 2007; Munshi‐South et al. 2016; Gortat et al. 2017; Lourenço et al. 2017). In contrast, urban populations of strong dispersers, such as birds, are not expected to exhibit losses of genetic diversity to the same degree (Johnson and Munshi‐South 2017). In agreement with this prediction, a cross‐species analysis of robust genetic data revealed consistently lower genetic diversity in urban populations of North American mammals, but not birds (Schmidt et al. 2020). Our contrasting results for the Eurasian coot may reflect two non‐exclusive processes. First, long‐term monitoring of marked birds has revealed negligible movement of individuals among urban populations and relatively limited immigration from non‐urban populations into old, well‐established urban populations (PM, unpublished data). Our study urban coot populations are likely self‐sustaining, and urban birds show high breeding site fidelity, which may promote strong genetic isolation and accelerate genetic drift. Second, the effect size for the reduction in allelic richness in urban populations was higher for immune genes (especially MHC‐I) than for neutral microsatellite markers, which may suggest a contribution of selection. This interpretation is consistent with our previous analysis of immunogenetic variation in a single urban coot population, which revealed negative associations of individual MHC diversity with fitness‐related traits, such as body condition, expression of putative sexual ornamentation, and reproduction (Pikus et al. 2022). Assuming that urban environments harbour less diverse pathogen communities (because many natural pathogens may be excluded from urban environments; Werner and Nunn 2020), the individual costs of high MHC diversity may outweigh the benefits associated with a broader MHC repertoire. This, in turn, could lead to a relaxation of balancing selection at the population level and a potentially adaptive reduction of immunogenetic variation. Empirical data on changes in the composition of coot pathogen communities along an urbanization gradient are required to test this hypothesis in future research.

Although we found a signature of immunogenetic differentiation between landscapes, the strongest differentiation was recorded among urban populations from different cities. Once again, we suggest that both neutral and selective processes could have contributed to this pattern. First, our previous analyses of microsatellite markers indicated that different cities in Poland were independently colonized by coots immigrating from nearby non‐urban areas (Minias et al. 2017). Consequently, initial differentiation among urban populations may have arisen through founder effects and increased over time through genetic drift. This scenario is consistent with the stronger microsatellite differentiation among urban populations than among non‐urban populations, as observed in the present study. On the other hand, urban populations exhibited significantly stronger differentiation at MHC‐II and TLR1LB compared to differentiation at microsatellites, which is indicative of selective processes. We suggest that individual cities may constitute unique ecosystems with distinct pathogen communities, which may facilitate adaptive immunogenetic differentiation among geographically isolated urban populations. At the same time, we acknowledge that the lack of data on pathogen pressure in urban and non‐urban coot populations represents a major limitation of our study, limiting the strength of our inferences. However, whole‐genome resequencing in the burrowing owl provided support for similar urbanization‐related processes, including independent urban colonization histories and founder effects resulting in loss of rare allelic variants (Mueller et al. 2018). As selection following urbanization is expected to act primarily on rare standing variation, the patterns of selection were predicted to be markedly variable between cities (Mueller et al. 2018). Consistently, continent‐scale genomic analyses of great tit *Parus major* populations revealed polygenic allele frequency shifts associated with the adaptation to urban environments, but haplotypes under selection were rarely shared across urban populations (Salmón et al. 2021). Together, these lines of evidence suggest that cities may function as evolutionary islands, with urban populations following largely independent evolutionary trajectories.

In conclusion, our study of replicated pairs of urban and non‐urban coot populations provides evidence for differentiation at both innate and adaptive immune genes. This differentiation was observed not only between urban and non‐urban landscapes but also among different urban populations. Comparisons with neutral microsatellite markers suggest that this pattern may have arisen through a combination of processes, including independent urban colonization and associated founder effects, drift in spatially and genetically isolated urban populations, and selection promoting changes in the immune gene repertoire following colonization of each urban site. Our results contribute to the understanding of mechanisms underlying wildlife urbanization and are consistent with the prediction that immune genes (particularly those directly involved in pathogen recognition) may be important targets of selection during urban colonization. While research on avian responses to urbanization has primarily focused on a handful of passerine land birds (such as tits Paridae), our study sheds new light on the processes of urban colonization in a non‐model non‐passerine species associated with urban aquatic ecosystems. We suggest that better mechanistic understanding of urbanization processes across diverse taxa may facilitate the conservation and management of urban wildlife. Our findings also suggest that considering immunogenetic variation may inform management decisions and help maintain biodiversity in urban landscapes. At the same time, we acknowledge that genotypic divergence and genotype–environment associations are insufficient to infer urban adaptation, which also requires empirical evidence that urban phenotypes are differentiated, heritable, and linked to fitness (Lambert et al. 2021). Further data on variation in infection traits and fitness among urban and non‐urban populations will be necessary to determine whether the observed immunogenetic differentiation reflects adaptation to urbanization‐related pathogen‐mediated selection.

## Funding

This work was financially supported by the research grant of the National Science Centre in Poland (2020/38/E/NZ8/00143).

## Ethics Statement

All procedures were approved by the Local Bioethical Commission for Experiments on Animals in Łódź (15/ŁB/2016 and 6/ŁB 157/2020).

## Conflicts of Interest

The authors declare no conflicts of interest.

## Supporting information


**Table S1:** Genetic diversity measures for 15 microsatellite markers across four pairs of urban and non‐urban populations of the Eurasian coot (*n* = 246 individuals). N_A_, number of alleles; H_e_, expected heterozygosity; H_o_, observed heterozygosity; F_IS_, inbreeding coefficient; F_null_, null alleles frequency.
**Table S2:** Differentiation at TLR1LB among landscapes and populations in the Eurasian coot. Differentiation was quantified using Jost's D based on allelic variants (below the diagonal) and amino acid variants (above the diagonal). Significant values are marked in bold.
**Table S3:** Differentiation at TLR3 among landscapes and populations in the Eurasian coot. Differentiation was quantified using Jost's D based on allelic variants (below the diagonal) and amino acid variants (above the diagonal). Significant values are marked in bold.
**Table S4:** Differentiation at TLR4 among landscapes and populations in the Eurasian coot. Differentiation was quantified using Jost's D based on allelic variants (below the diagonal) and amino acid variants (above the diagonal). Significant values are marked in bold.
**Table S5:** Differentiation at TLR5 among landscapes and populations in the Eurasian coot. Differentiation was quantified using Jost's D based on allelic variants (below the diagonal) and amino acid variants (above the diagonal). Significant values are marked in bold.
**Table S6:** Comparisons of TLR allelic differentiation among Eurasian coot populations: (i) between urban and non‐urban landscape (between_landsc), (ii) within urban landscape (within_urban_landsc) and (iii) within non‐urban landscape (reference category represented by the intercept). Population differentiation was quantified using pairwise Jost's D values. Significant predictors are marked in bold.
**Table S7:** Comparisons of TLR amino acid differentiation among Eurasian coot populations: (i) between urban and non‐urban landscape (between_landsc), (ii) within urban landscape (within_urban_landsc) and (iii) within non‐urban landscape (reference category represented by the intercept). Population differentiation was quantified using pairwise Jost's D values. Significant predictors are marked in bold.
**Table S8:** Associations between landscape type and the occurrence of individual TLR amino acid variants in the Eurasian coot. TLR4 was excluded from the analysis, as none amino acid variant showed 10%–90% frequency. Positive estimates indicate a higher probability of variant occurrence in urban populations. Significant predictors are marked in bold.
**Table S9:** Comparisons of MHC allelic differentiation among Eurasian coot populations: (i) between urban and non‐urban landscape (between_landsc), (ii) within urban landscape (within_urban_landsc) and (iii) within non‐urban landscape (reference category represented by the intercept). Population differentiation was quantified using pairwise Jost's D values. Significant predictors are marked in bold.
**Table S10:** Associations between landscape type and the occurrence of individual MHC functional supertypes in the Eurasian coot. Negative estimates indicate a lower probability of supertype occurrence in urban populations. Significant predictors are marked in bold.
**Table S11:** Differentiation at 15 neutral microsatellites markers among landscapes and populations in the Eurasian coot. Differentiation was quantified using pairwise Jost's D values. Significant values are marked in bold.
**Table S12:** Comparisons of microsatellite differentiation among Eurasian coot populations: (i) between urban and non‐urban landscape (between_landsc), (ii) within urban landscape (within_urban_landsc) and (iii) within non‐urban landscape (reference category represented by the intercept). Population differentiation was quantified using pairwise Jost's D values. Significant predictors are marked in bold.
**Table S13:** Comparisons of population differentiation at immunogenetic and neutral (microsatellite) markers among Eurasian coot populations. Population differentiation was quantified using pairwise Jost's D values. Population‐pair identity was entered as a random effect. Differentiation at microsatellite markers was used as a reference category represented by the intercept. Significant predictors are marked in bold.
**Figure S1:** Results of permutation tests comparing allelic richness across four TLR loci between urban and non‐urban landscape in the Eurasian coot. Grey bars show the distribution of permuted differences (*n* = 1000 permutations), while the red line indicates the observed difference between landscapes.
**Figure S2:** Results of permutation tests comparing allelic richness at MHC‐I (A) and MHC‐II (B) between urban and non‐urban landscape in the Eurasian coot. Grey bars show the distribution of permuted differences (*n* = 1000 permutations), while the red line indicates the observed difference between landscapes.
**Figure S3:** Population differentiation at 15 neutral microsatellite markers among urban populations, between urban and non‐urban populations, and among non‐urban in the Eurasian coot. Central line—mean, box—SE, whiskers—95% confidence interval.

## Data Availability

All data used in this study have been deposited in Dryad (doi: 10.5061/dryad.k3j9kd5r8). All novel sequences have been deposited in GenBank (QB247319‐QB248398).
