# Supplementary material for: Cities as Evolutionary Islands: Colonization of Isolated Urban Habitats Promotes Immunogenetic Differentiation in a Common Waterbird
Source: Evol Appl. 2026 Nov 11;19(11):e70363. doi: 10.1111/eva.70363 (PMC13755681; doi:10.1111/eva.70363)

Supporting Information

Cities as evolutionary islands: colonization of isolated urban habitats promotes immunogenetic differentiation in a common waterbird

Piotr Minias[1] | Joanna Drzewińska-Chańko[1] | Jan Jedlikowski[2] | Amelia Chyb[1] | Radosław Włodarczyk[1]

[1]University of Lodz, Faculty of Biology and Environmental Protection, Department of Biodiversity Studies and Bioeducation, Banacha 1/3, 90-237 Lodz, Poland;

[2]University of Warsaw, Faculty of Biology, Biological and Chemical Research Centre, Żwirki i Wigury 101, 02-089, Warsaw, Poland;

Correspondence

Piotr Minias (pminias@op.pl)

TABLE S1 | Genetic diversity measures for 15 microsatellite markers across four pairs of urban and non-urban populations of the Eurasian coot (n = 246 individuals). N_A – number of alleles; H_e – expected heterozygosity; H_o – observed heterozygosity; F_{IS} – inbreeding coefficient; F_{null} – null alleles frequency.

Marker	N_A	H_e	H_o	F_{IS}	F_{null}
Fa1	12	0.72	0.69	0.04	0.03
Fa2	20	0.90	0.83	0.09	0.02
Fa3	22	0.89	0.85	0.04	-0.02
Fa9	7	0.52	0.51	0.01	0.04
Fa10	30	0.94	0.84	0.10	0.05
Fa11	8	0.65	0.53	0.18	0.02
Fa13	5	0.50	0.54	-0.09	0.00
Fa14	21	0.89	0.86	0.04	0.05
Fa15	16	0.85	0.87	-0.03	0.09
Fa16	33	0.87	0.80	0.07	-0.06
B106	19	0.82	0.81	0.01	0.00
KiRa9	7	0.50	0.52	-0.04	-0.02
KiRa10	13	0.77	0.75	0.03	0.02
KiRa16	54	0.97	0.94	0.03	0.01
Tm27	21	0.85	0.83	0.03	0.01

TABLE S2 | Differentiation at TLR1LB among landscapes and populations in the Eurasian coot. Differentiation was quantified using Jost's D based on allelic variants (below the diagonal) and amino acid variants (above the diagonal). Significant values are marked in bold.

Landscape/ population		Urban				Non-urban			
		Katowice	Łódź	Poznań	Warszawa	Katowice	Łódź	Poznań	Warszawa
Urban	Katowice	-	0.032	0.112	**0.153**	0.120	-0.035	-0.071	-0.096
	Łódź	0.041	-	**0.225**	0.020	**0.198**	**0.122**	-0.013	0.013
	Poznań	0.141	**0.326**	-	**0.254**	0.052	0.036	**0.133**	**0.159**
	Warszawa	**0.156**	0.121	**0.406**	-	**0.190**	**0.217**	0.089	**0.160**
Non-urban	Katowice	**0.264**	**0.317**	0.173	**0.414**	-	0.112	0.156	**0.229**
	Łódź	0.019	**0.205**	0.076	**0.320**	**0.201**	-	-0.011	-0.047
	Poznań	-0.055	-0.026	0.071	0.133	0.195	0.016	-	-0.076
	Warszawa	-0.096	0.023	0.127	**0.215**	**0.369**	0.016	-0.123	-

TABLE S3 | Differentiation at TLR3 among landscapes and populations in the Eurasian coot. Differentiation was quantified using Jost's D based on allelic variants (below the diagonal) and amino acid variants (above the diagonal). Significant values are marked in bold.

Landscape/ population		Urban				Non-urban			
		Katowice	Łódź	Poznań	Warszawa	Katowice	Łódź	Poznań	Warszawa
Urban	Katowice	-	0.034	0.009	**0.121**	0.025	-0.018	0.013	0.013
	Łódź	0.075	-	-0.022	0.029	-0.012	-0.006	0.016	-0.023
	Poznań	0.032	0.109	-	0.013	-0.026	-0.024	0.027	-0.033
	Warszawa	0.088	-0.039	0.073	-	0.042	0.057	**0.153**	0.022
Non-urban	Katowice	0.000	0.014	-0.020	0.020	-	-0.011	0.013	-0.029
	Łódź	-0.043	0.010	0.006	0.015	-0.018	-	0.026	-0.031
	Poznań	-0.028	-0.013	-0.016	0.007	-0.073	-0.032	-	0.019
	Warszawa	-0.001	0.054	-0.036	0.009	-0.001	-0.085	-0.018	-

TABLE S4 | Differentiation at TLR4 among landscapes and populations in the Eurasian coot. Differentiation was quantified using Jost's D based on allelic variants (below the diagonal) and amino acid variants (above the diagonal). Significant values are marked in bold.

Landscape/ population		Urban				Non-urban			
		Katowice	Łódź	Poznań	Warszawa	Katowice	Łódź	Poznań	Warszawa
Urban	Katowice	-	-0.001	-0.001	0.004	-0.001	0.000	-0.001	0.001
	Łódź	-0.002	-	-0.001	0.005	-0.001	0.000	-0.001	0.001
	Poznań	-0.005	-0.004	-	0.003	-0.001	-0.001	-0.001	-0.001
	Warszawa	-0.001	0.002	-0.001	-	0.003	0.003	0.003	0.001
Non-urban	Katowice	-0.005	-0.001	-0.005	-0.001	-	-0.001	-0.001	-0.001
	Łódź	0.001	-0.008	-0.004	0.003	0.002	-	-0.001	-0.002
	Poznań	0.003	-0.001	-0.003	0.005	0.004	-0.007	-	-0.001
	Warszawa	-0.001	0.004	-0.004	0.001	-0.001	0.000	-0.005	-

TABLE S5 | Differentiation at TLR5 among landscapes and populations in the Eurasian coot. Differentiation was quantified using Jost's D based on allelic variants (below the diagonal) and amino acid variants (above the diagonal). Significant values are marked in bold.

Landscape/ population		Urban				Non-urban			
		Katowice	Łódź	Poznań	Warszawa	Katowice	Łódź	Poznań	Warszawa
Urban	Katowice	-	-0.007	0.010	-0.020	-0.028	0.003	0.011	-0.001
	Łódź	-0.002	-	-0.010	0.028	-0.004	0.046	**0.075**	-0.005
	Poznań	0.034	-0.012	-	0.019	0.014	0.018	0.054	-0.020
	Warszawa	-0.018	0.026	0.053	-	-0.013	-0.002	-0.013	0.009
Non-urban	Katowice	-0.026	-0.012	0.023	-0.011	-	-0.008	0.022	0.000
	Łódź	0.001	0.049	0.037	0.005	-0.004	-	0.029	0.028
	Poznań	-0.012	0.054	0.059	-0.010	0.014	0.018	-	0.037
	Warszawa	-0.011	-0.007	0.002	0.004	-0.008	0.025	0.010	-

TABLE S6 | Comparisons of TLR allelic differentiation among Eurasian coot populations: *i*) between urban and non-urban landscape (between_landsc), *ii*) within urban landscape (within_urban_landsc) and *iii*) within non-urban landscape (reference category represented by the intercept). Population differentiation was quantified using pairwise Jost's D values. Significant predictors are marked in bold.

Locus	Comparison	Estimate	SE	t	p
All loci	Intercept	0.002	0.006	0.33	0.742
	D_between_landsc	0.009	0.006	1.41	0.171
	**D_within_urban_landsc**	**0.023**	**0.008**	**2.93**	**0.007**
TLR1LB	Intercept	0.112	0.062	1.82	0.081
	D_between_landsc	0.024	0.072	0.33	0.744
	D_within_urban_landsc	0.086	0.087	0.99	0.334
TLR3	**Intercept**	**-0.038**	**0.014**	**2.75**	**0.011**
	**D_between_landsc**	**0.036**	**0.016**	**2.26**	**0.033**
	**D_within_urban_landsc**	**0.094**	**0.019**	**4.84**	**<0.001**
TLR4	Intercept	-0.001	0.001	0.80	0.433
	D_between_landsc	0.000	0.002	0.10	0.923
	D_within_urban_landsc	-0.001	0.002	0.32	0.750
TLR5	Intercept	0.010	0.010	0.90	0.378
	D_between_landsc	0.000	0.012	0.01	0.993
	D_within_urban_landsc	0.004	0.014	0.40	0.766

TABLE S7 | Comparisons of TLR amino acid differentiation among Eurasian coot populations: *i*) between urban and non-urban landscape (between_landsc), *ii*) within urban landscape (within_urban_landsc) and *iii*) within non-urban landscape (reference category represented by the intercept). Population differentiation was quantified using pairwise Jost's D values. Significant predictors are marked in bold.

Locus	Comparison	Estimate	SE	t	p
All loci	Intercept	0.007	0.006	1.27	0.217
	D_between_landsc	0.006	0.006	0.91	0.374
	D_within_urban_landsc	0.014	0.008	1.83	0.079
TLR1LB	Intercept	0.061	0.042	1.42	0.165
	D_between_landsc	0.019	0.050	0.39	0.703
	D_within_urban_landsc	0.072	0.060	1.21	0.239
TLR3	Intercept	-0.002	0.017	0.12	0.902
	D_between_landsc	0.016	0.021	0.80	0.434
	D_within_urban_landsc	0.033	0.025	1.33	0.196
TLR4	Intercept	-0.001	0.001	1.63	0.117
	D_between_landsc	0.001	0.001	1.58	0.105
	**D_within_urban_landsc**	**0.003**	**0.001**	**2.63**	**0.015**
TLR5	Intercept	0.018	0.010	1.80	0.084
	D_between_landsc	-0.010	0.012	0.77	0.450
	D_within_urban_landsc	-0.015	0.014	1.04	0.309

TABLE S8 | | Associations between landscape type and the occurrence of individual TLR amino acid variants in the Eurasian coot. TLR4 was excluded from the analysis, as none amino acid variant showed 10-90% frequency. Positive estimates indicate a higher probability of variant occurrence in urban populations. Significant predictors are marked in bold.

Locus	Predictor	Estimate	SE	z	p
TLR1LB	Intercept	-0.475	0.308	-1.54	0.123
	aa_TLR1LB*01	0.621	0.330	1.88	0.060
	**aa_TLR1LB*02**	**0.751**	**0.335**	**2.24**	**0.025**
	aa_TLR1LB*03	-0.170	0.349	-0.49	0.627
	aa_TLR1LB*14	0.133	0.437	0.30	0.762
TLR3	Intercept	0.548	0.442	1.24	0.215
	aa_TLR3*01	-0.497	0.420	-1.18	0.237
	aa_TLR3*03	-0.090	0.346	-0.26	0.795
TLR5	(Intercept)	-0.502	0.437	-1.15	0.250
	aa_TLR5*01	0.290	0.401	0.72	0.470
	aa_TLR5*04	0.347	0.486	0.71	0.475
	aa_TLR5*05	0.630	0.372	1.69	0.091
	aa_TLR5*16	0.549	0.349	1.57	0.116

TABLE S9 | Comparisons of MHC allelic differentiation among Eurasian coot populations: *i*) between urban and non-urban landscape (between_landsc), *ii*) within urban landscape (within_urban_landsc) and *iii*) within non-urban landscape (reference category represented by the intercept). Population differentiation was quantified using pairwise Jost's D values. Significant predictors are marked in bold.

Locus	Comparison	Estimate	SE	t	p
MHC-I	Intercept	0.000	0.02	0.00	1.000
	D_between_landsc	0.029	0.018	1.63	0.115
	**D_within_urban_landsc**	**0.067**	**0.021**	**3.09**	**0.005**
MHC-II	Intercept	0.040	0.047	0.86	0.401
	**D_between_landsc**	**0.122**	**0.055**	**2.21**	**0.036**
	**D_within_urban_landsc**	**0.279**	**0.066**	**4.20**	**<0.001**

TABLE S10 | Associations between landscape type and the occurrence of individual MHC functional supertypes in the Eurasian coot. Negative estimates indicate a lower probability of supertype occurrence in urban populations. Significant predictors are marked in bold.

Locus	Predictor	Estimate	SE	z	p
MHC-I	Intercept	0.987	0.866	1.14	0.255
	MHC-I-ST1	-0.533	0.313	-1.70	0.089
	MHC-I-ST2	0.663	0.351	1.89	0.059
	MHC-I-ST5	-0.235	0.362	-0.65	0.516
	MHC-I-ST6	-0.377	0.286	-1.32	0.188
	MHC-I-ST7	-0.095	0.316	-0.30	0.764
	MHC-I-ST8	0.324	0.298	1.09	0.276
	MHC-I-ST9	0.190	0.426	0.45	0.656
	**MHC-I-ST10**	**-1.016**	**0.453**	**-2.24**	**0.025**
	MHC-I-ST12	0.192	0.291	0.66	0.508
	MHC-I-ST14	0.081	0.282	0.29	0.774
	MHC-I-ST15	-0.461	0.345	-1.34	0.181
	MHC-I-ST17	0.320	0.279	1.15	0.252
	MHC-I-ST18	0.055	0.319	0.17	0.864
	MHC-I-ST19	-0.042	0.279	-0.15	0.880
MHC-II	(Intercept)	0.515	0.460	1.12	0.263
	MHC-II-ST1	-0.018	0.316	-0.06	0.955
	MHC-II-ST2	-0.300	0.307	-0.98	0.327
	**MHC-II-ST3**	**-1.018**	**0.345**	**-2.95**	**0.003**
	MHC-II-ST4	-0.263	0.351	-0.75	0.453
	MHC-II-ST6	0.067	0.305	0.22	0.826
	MHC-II-ST8	-0.191	0.384	-0.50	0.619
	MHC-II-ST10	0.193	0.445	0.43	0.665

TABLE S11 | Differentiation at 15 neutral microsatellites markers among landscapes and populations in the Eurasian coot. Differentiation was quantified using pairwise Jost's D values. Significant values are marked in bold.

Landscape/ population		Urban				Non-urban			
		Katowice	Łódź	Poznań	Warszawa	Katowice	Łódź	Poznań	Warszawa
Urban	Katowice	-	na	na	na	na	na	na	na
	Łódź	**0.055**	-	na	na	na	na	na	na
	Poznań	**0.044**	**0.047**	-	na	na	na	na	na
	Warszawa	**0.080**	**0.095**	**0.062**	-	na	na	na	na
Non-urban	Katowice	**0.026**	**0.023**	0.003	**0.082**	-	na	na	na
	Łódź	**0.037**	0.011	-0.001	**0.068**	0.001	-	na	na
	Poznań	0.014	**0.030**	0.005	**0.071**	0.011	-0.001	-	na
	Warszawa	**0.035**	**0.023**	-0.003	**0.076**	-0.010	0.000	0.002	-

TABLE S12 | Comparisons of microsatellite differentiation among Eurasian coot populations: *i*) between urban and non-urban landscape (between_landsc), *ii*) within urban landscape (within_urban_landsc) and *iii*) within non-urban landscape (reference category represented by the intercept). Population differentiation was quantified using pairwise Jost's D values. Significant predictors are marked in bold.

Comparison	Estimate	SE	t	p
Intercept	0.001	0.010	0.05	0.960
**D_between_landsc**	**0.031**	**0.011**	**2.68**	**0.013**
**D_within_urban_landsc**	**0.063**	**0.014**	**4.58**	**<0.001**

TABLE S13 | Comparisons of population differentiation at immunogenetic and neutral (microsatellite) markers among Eurasian coot populations. Population differentiation was quantified using pairwise Jost's D values. Population-pair identity was entered as a random effect. Differentiation at microsatellite markers was used as a reference category represented by the intercept. Significant predictors are marked in bold.

Marker	Estimate	SE	t	p
**Intercept**	**0.032**	**0.016**	**2.01**	**0.046**
MHC-I	-0.001	0.020	-0.04	0.969
**MHC-II**	**0.138**	**0.020**	**6.73**	**<0.001**
**TLR1LB**	**0.113**	**0.020**	**5.51**	**<0.001**
TLR3	-0.028	0.020	-1.39	0.167
TLR4	-0.033	0.020	-1.60	0.111
TLR5	-0.022	0.020	-1.06	0.293

FIGURE S1 | Results of permutation tests comparing allelic richness across four TLR loci between urban and non-urban landscape in the Eurasian coot. Grey bars show the distribution of permuted differences (n = 1000 permutations), while the red line indicates the observed difference between landscapes.

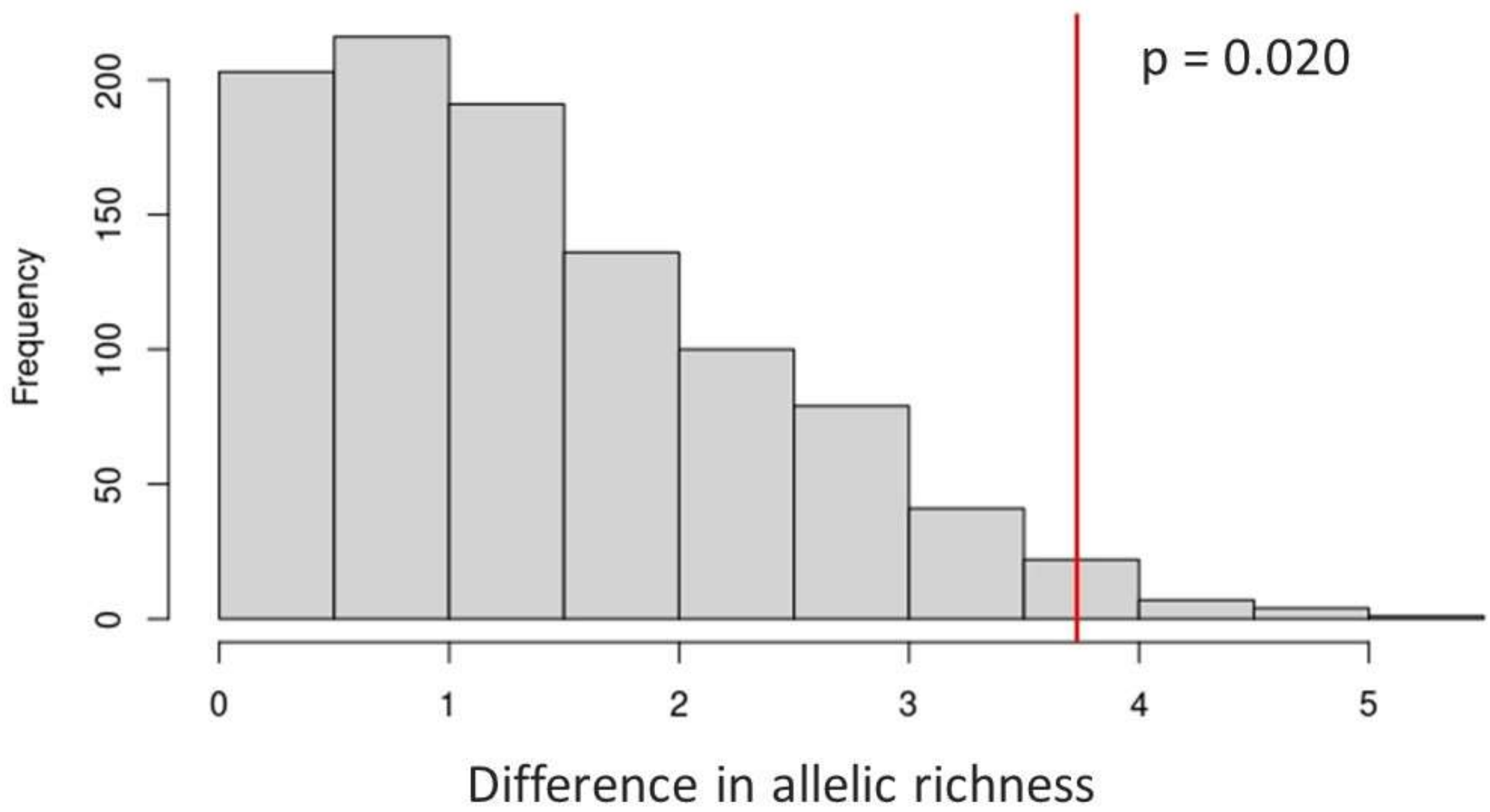

FIGURE S2 | Results of permutation tests comparing allelic richness at MHC-I (A) and MHC-II (B) between urban and non-urban landscape in the Eurasian coot. Grey bars show the distribution of permuted differences (n = 1000 permutations), while the red line indicates the observed difference between landscapes.

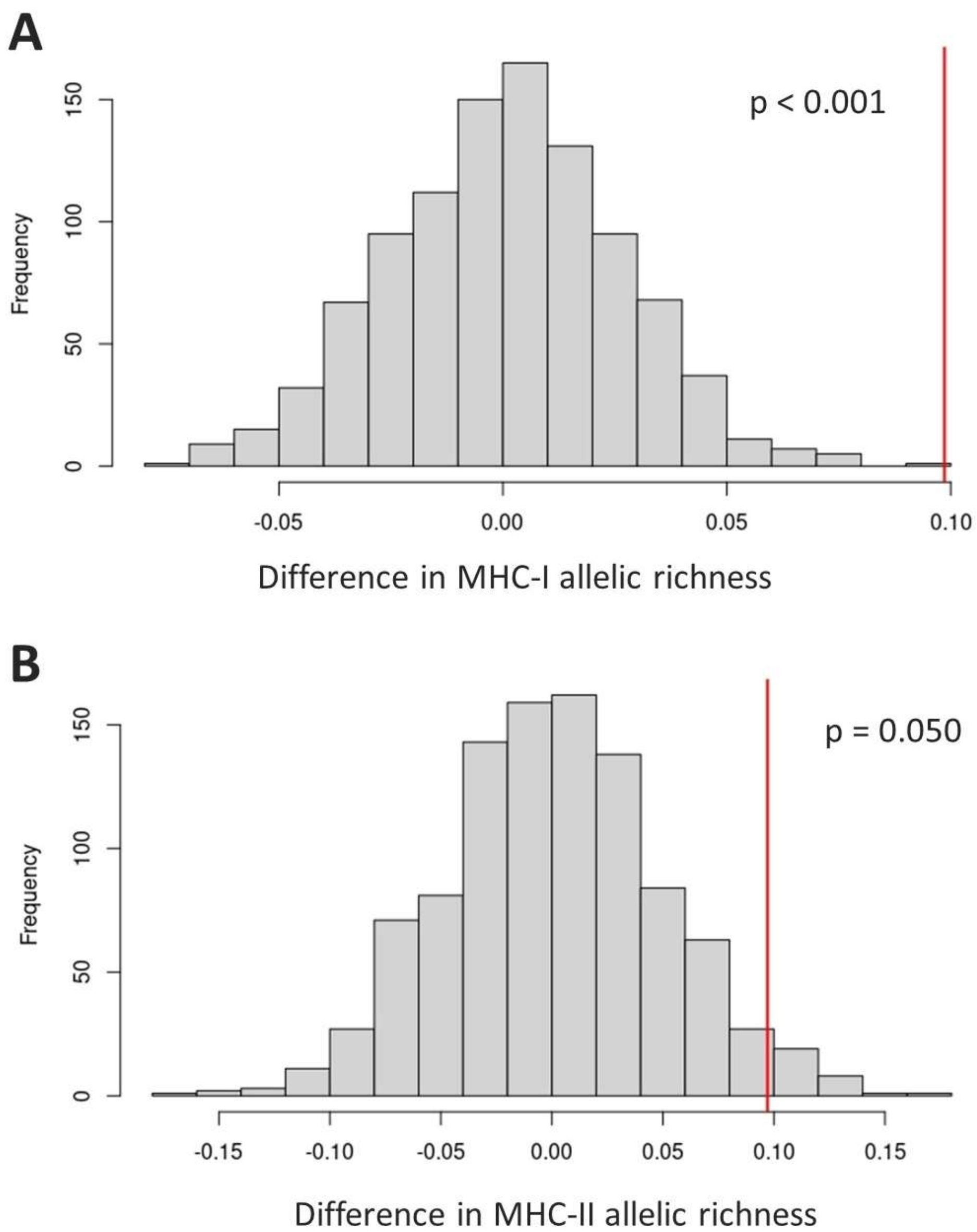

FIGURE S3 | Population differentiation at 15 neutral microsatellite markers among urban populations, between urban and non-urban populations, and among non-urban in the Eurasian coot. Central line – mean, box – SE, whiskers – 95% confidence interval.

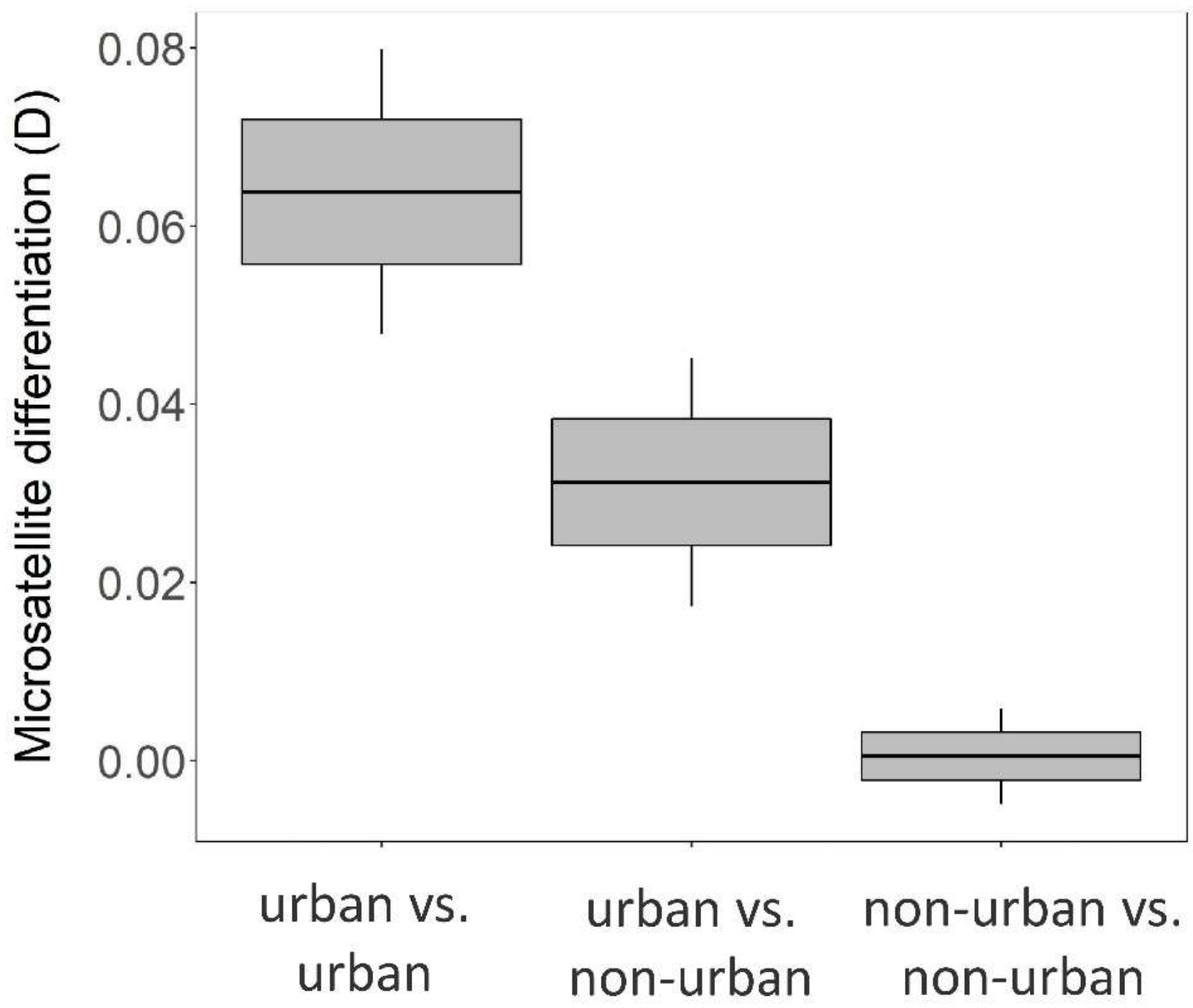

Supplement: Supplementary file 1 [file EVA-19-e70363-s001.pdf]
